# MEK5/ERK5 signaling inhibition increases colon cancer cell sensitivity to 5-fluorouracil through a p53-dependent mechanism

**DOI:** 10.18632/oncotarget.9107

**Published:** 2016-04-29

**Authors:** Diane M. Pereira, André E. S. Simões, Sofia E. Gomes, Rui E. Castro, Tânia Carvalho, Cecília M. P. Rodrigues, Pedro M. Borralho

**Affiliations:** ^1^ Research Institute for Medicines (iMed.ULisboa), Faculty of Pharmacy, Universidade de Lisboa, Lisbon, Portugal; ^2^ Histology and Comparative Pathology Laboratory, Instituto de Medicina Molecular, Lisbon, Portugal

**Keywords:** MEK5/ERK5, p53, 5-fluorouracil, apoptosis, chemosensitization

## Abstract

The MEK5/ERK5 signaling pathway is emerging as an important contributor to colon cancer onset, progression and metastasis; however, its relevance to chemotherapy resistance remains unknown. Here, we evaluated the impact of the MEK5/ERK5 cascade in colon cancer cell sensitivity to 5-fluorouracil (5-FU). Increased ERK5 expression was correlated with poor overall survival in colon cancer patients. In colon cancer cells, 5-FU exposure impaired endogenous KRAS/MEK5/ERK5 expression and/or activation. In turn, MEK5 constitutive activation reduced 5-FU-induced cytotoxicity. Using genetic and pharmacological approaches, we showed that ERK5 inhibition increased caspase-3/7 activity and apoptosis following 5-FU exposure. Mechanistically, this was further associated with increased p53 transcriptional activation of *p21* and *PUMA*. In addition, ERK5 inhibition increased the response of HCT116 p53^+/+^ cells to 5-FU, but failed to sensitize HCT116 p53^−/−^ cells to the cytotoxic effects of this chemotherapeutic agent, suggesting a p53-dependent axis mediating 5-FU sensitization. Finally, ERK5 inhibition using XMD8-92 was shown to increase the antitumor effects of 5-FU in a murine subcutaneous xenograft model, enhancing apoptosis while markedly reducing tumor growth. Collectively, our results suggest that ERK5-targeted in hibition provides a promising therapeutic approach to overcome resistance to 5-FU-based chemotherapy and improve colon cancer treatment.

## INTRODUCTION

Over the last five decades, the antimetabolite 5-fluorouracil (5-FU) has been the mainstay of colon cancer therapy, both in adjuvant and metastatic settings [1]. Moreover, progress in the treatment of colon cancer has been achieved after the introduction of new cytotoxic and targeted agents to the existing 5-FU/leucovorin standard [1, 2]. However, intrinsic and acquired resistance remains a major setback to 5-FU clinical efficacy [3]. Up to 40% of patients receiving 5-FU-based adjuvant chemotherapy following potentially curative resection of stage II and III colon tumors experience recurrence or die within 8 years of follow-up [4]. Also, while response rates to current 5-FU combination therapies may be as high as 50%, the median overall survival for patients with metastatic colon cancer is still limited to, at best, 30 months [5–8]. Therefore, a deeper understanding of the signaling pathways mediating 5-FU resistance will importantly nourish the identification of new therapeutic targets and combination strategies that could ultimately translate into improved outcomes for colon cancer patients.

The mitogen-activated protein kinase (MAPK) cascades are among the most frequently deregulated signaling pathways in human cancer [9]. Of particular interest, the extracellular signal-regulated kinase 5 (ERK5 or BMK1), together with its upstream activator MAPK kinase 5 (MEK5), mediate the most recently described MAPK pathway [10]. Importantly, aberrant MEK5/ERK5 signaling has been reported in several types of human cancer, including colon cancer [11, 12], and its association with increased cell proliferation, tumor angiogenesis and metastasis is becoming increasingly recognized [13, 14]. Notably, we have recently demonstrated that MEK5 and ERK5 expression is increased in human colon adenomas and adenocarcinomas, and that ERK5 overexpression correlates with increased invasion, as well as with presence of lymph node and distant metastasis [11]. In addition, MEK5 overactivation has also been associated with colon cancer stage progression [12]. Nevertheless, the full extent of cellular and molecular mechanisms by which the MEK5/ERK5 cascade contributes to colon cancer pathogenesis remains unclear, as does the relevance of this signaling pathway to chemotherapy response.

The p53 tumor suppressor is a major determinant of the balance between cell cycle arrest and apoptosis following 5-FU exposure [15]. In turn, loss of p53 function is the most common mechanism by which tumor cells evade apoptosis [16], and was shown to abolish the anticancer effects of 5-FU in colon cancer cells and animal models [17, 18]. Although the predictive value of p53 status as a marker for 5-FU response remains controversial, several clinical studies have suggested that loss of p53 function may correlate with increased tumor resistance to 5-FU-based therapy [19–21]. Interestingly, active ERK5 was recently demonstrated as being involved in the suppression of p53 function [22, 23]. However, no relationship has been established between MEK5/ERK5 dysregulation and 5-FU resistance thus far.

In the present study, we aimed to evaluate the role of MEK5/ERK5 signaling pathway in the context of colon cancer cell sensitivity to 5-FU. Altogether, our results uncover an important link between the MEK5/ERK5 cascade and p53-dependent apoptosis triggered by 5-FU, where ERK5-targeted inhibition arises as a promising therapeutic approach for colon cancer treatment and chemosensitization.

## RESULTS

### High ERK5 expression is associated with poor prognosis in colon cancer patients

**Figure 1:**
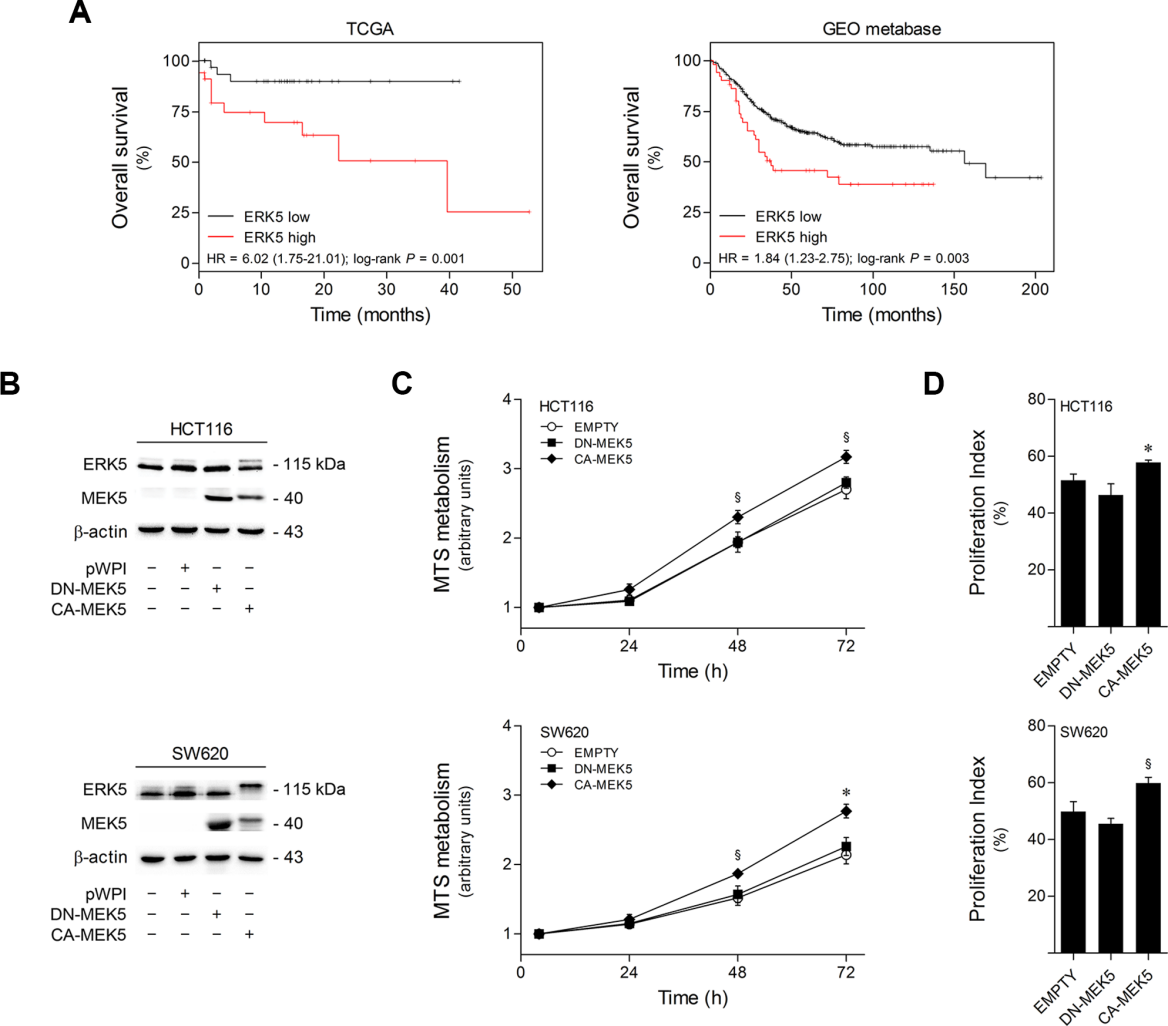
High ERK5 expression in colon cancer correlates with poor patient survival, and MEK5 constitutive activation increases colon cell proliferation (**A**) Kaplan-Meier analysis of overall survival in colorectal patients from TCGA database (left panel, *n* = 151) and GEO metabase (right panel, *n* = 482). Patients were grouped according to survival risk based on tumor ERK5 mRNA expression levels. Low- (TCGA, *n* = 84; GEO metabase, *n* = 431) and high-ERK5 expression subsets (TCGA, *n* = 67; GEO metabase, *n* = 51) are shown in black and red, respectively. *P*-values were obtained using log-rank tests. (**B**) HCT116 and SW620 cells were transduced with lentiviral particles carrying pWPI-eGFP expression constructs encoding DN-MEK5, CA-MEK5, or EMPTY vector controls, followed by fluorescence-activated cell sorting to generate stable cell lines. MEK5 overexpression and ERK5 differential activation status were confirmed by immunoblotting. (**C**) The growth profiles of HCT116 and SW620 cells stably expressing DN-MEK5 and CA-MEK5, and empty controls, were monitored by MTS metabolism assay at 4, 24, 48 and 72 h after plating. (**D**) Proliferation indexes of HCT116 and SW620 cells stably expressing DN-MEK5 and CA-MEK5, and empty controls, were determined according to cell cycle distribution of exponentially growing populations according to the following formula: Proliferation index (%) =% of cells in S + G2 + M phases. Results are expressed as mean ± SEM of 5 independent experiments. ^§^*p <* 0.05 and *p < 0.01 from EMPTY cells.

To clarify the clinical relevance of ERK5 in colon cancer, the prognostic value of ERK5 mRNA expression was analyzed in two independent datasets of colon cancer patients using the SurvExpress web resource [24]. RNA sequencing and microarray data were respectively obtained from the TCGA database (151 samples), and from the colon cancer GEO metabase (482 samples). Kaplan-Meier curves showed that high ERK5 mRNA expression correlates with worse overall survival in both cohorts, as compared with patients with low ERK5 expression (log-rank *p* = 0.001 for TCGA and *p* = 0.003 for the GEO metabase) (Figure 1A). Importantly, these results indicate that increased ERK5 expression may be a significant marker of poor prognosis in colon cancer.

### MEK5/ERK5 constitutive activation promotes colon cancer cell proliferation

To define the functional role of ERK5-mediated signaling on colon cancer malignant features, we developed HCT116 and SW620-derived cell lines with differential MEK5/ERK5 activation. Constitutively active (CA) and dominant negative (DN) forms of MEK5 were used to induce or block ERK5 activation, respectively (Figure 1B). Resulting CA-MEK5 and DN-MEK5-expressing cell lines were produced by lentiviral transduction, followed by sorting of stably transduced cells. Empty vector-expressing cells were used as controls.

Next, we investigated the effects of ERK5 differential activation in colon cancer cell proliferation. Cell growth profiles showed that ERK5 overactivation by CA-MEK5 significantly increased HCT116 and SW620 cell proliferation by up to 20% (*p* < 0.05) and 30% (*p* < 0.01) at 72 h, respectively, compared to empty vector control cells (Figure 1C). Similarly, cell cycle analysis revealed that upon MEK5 constitutive activation the proliferation index of HCT116 and SW620 cells was increased by 15% (*p* < 0.01) and 20% (*p* < 0.05), respectively, as compared to empty vector control cells (Figure 1D). Collectively, these results suggest that MEK5/ERK5 signaling overactivation increases the proliferation rate of HCT116 and SW620 colon cancer cells.

### 5-FU impairs KRAS/MEK5/ERK5 signaling in colon cancer cells

**Figure 2:**
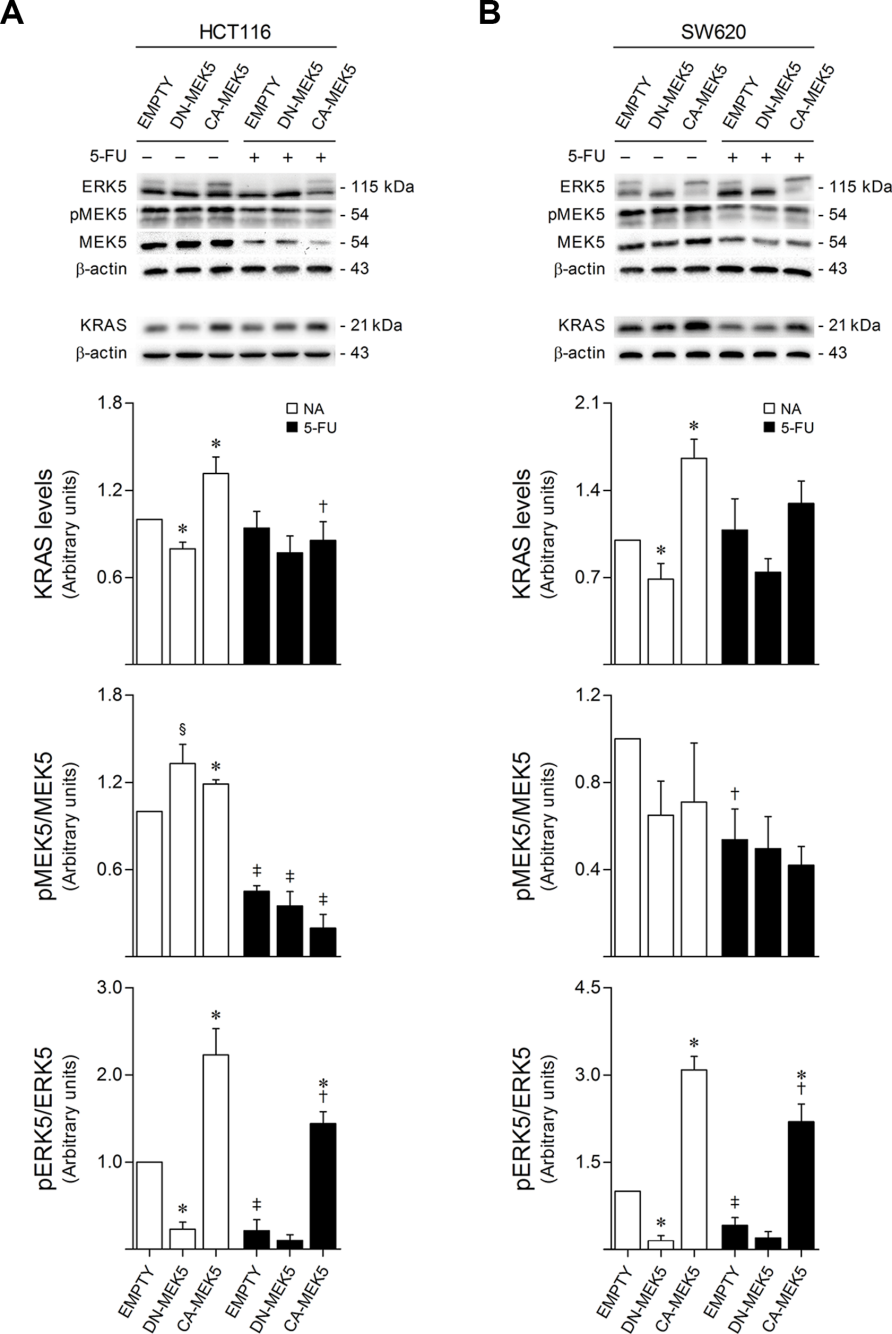
5-FU exposure reduces KRAS/MEK5/ERK5 protein expression and activation HCT116 (**A**) and SW620 (**B**) cells expressing DN-MEK5 or CA-MEK5, and empty controls, were exposed to 8 or 100 μM 5-FU, respectively. DMSO was used as vehicle control. At 72 h after treatment, cells were harvested for total protein extraction. Protein steady-state levels were evaluated by western blot. Representative blots are shown. Results are expressed as mean ± SEM fold-change from vehicle control EMPTY cells, of at least 3 independent experiments. ^§^*p <* 0.05 and *p < 0.01 from EMPTY cells; ^†^*p <* 0.05 and ^‡^*p <* 0.01 from respective vehicle control cells.

To determine the effects of 5-FU treatment in KRAS/MEK5/ERK5 signaling, HCT116 and SW620 cells were exposed to 8 and 100 μM 5-FU, respectively, for 72 h. Interestingly, CA-MEK5 and DN-MEK5 stable overexpression respectively led to a significant increase and decrease in KRAS protein steady-state levels, compared to empty vector control cells (*p* < 0.01). In addition, steady-state levels of KRAS protein were decreased upon 5-FU exposure in both HCT116 and SW620 cells expressing CA-MEK5, compared to corresponding vehicle treated cells (*p <* 0.05 in HCT116 cells) (Figure 2A and 2B, upper panel). Moreover, while no significant differences were detected in MEK5 protein steady-state levels, 5-FU treatment negatively modulated the levels of endogenous MEK5 activation in both colon cancer cell models (*p <* 0.01 in HCT116 cells) (Figure 2A and 2B, middle panel). Consistently, endogenous levels of ERK5 activation were also significantly reduced following 5-FU treatment in both HCT116 and SW620 cells stably overexpressing CA-MEK5 (*p <* 0.05), as well as in empty vector control cells (*p <* 0.01) (Figure 2A and 2B, lower panel). These results uncover a downregulating effect of 5-FU towards the KRAS/MEK5/ERK5 cascade, suggesting that inhibition of signaling through this pathway may be a major determinant of tumor cell response to 5-FU.

### MEK5/ERK5 signaling inhibition increases HCT116 cell sensitivity to 5-FU

**Figure 3:**
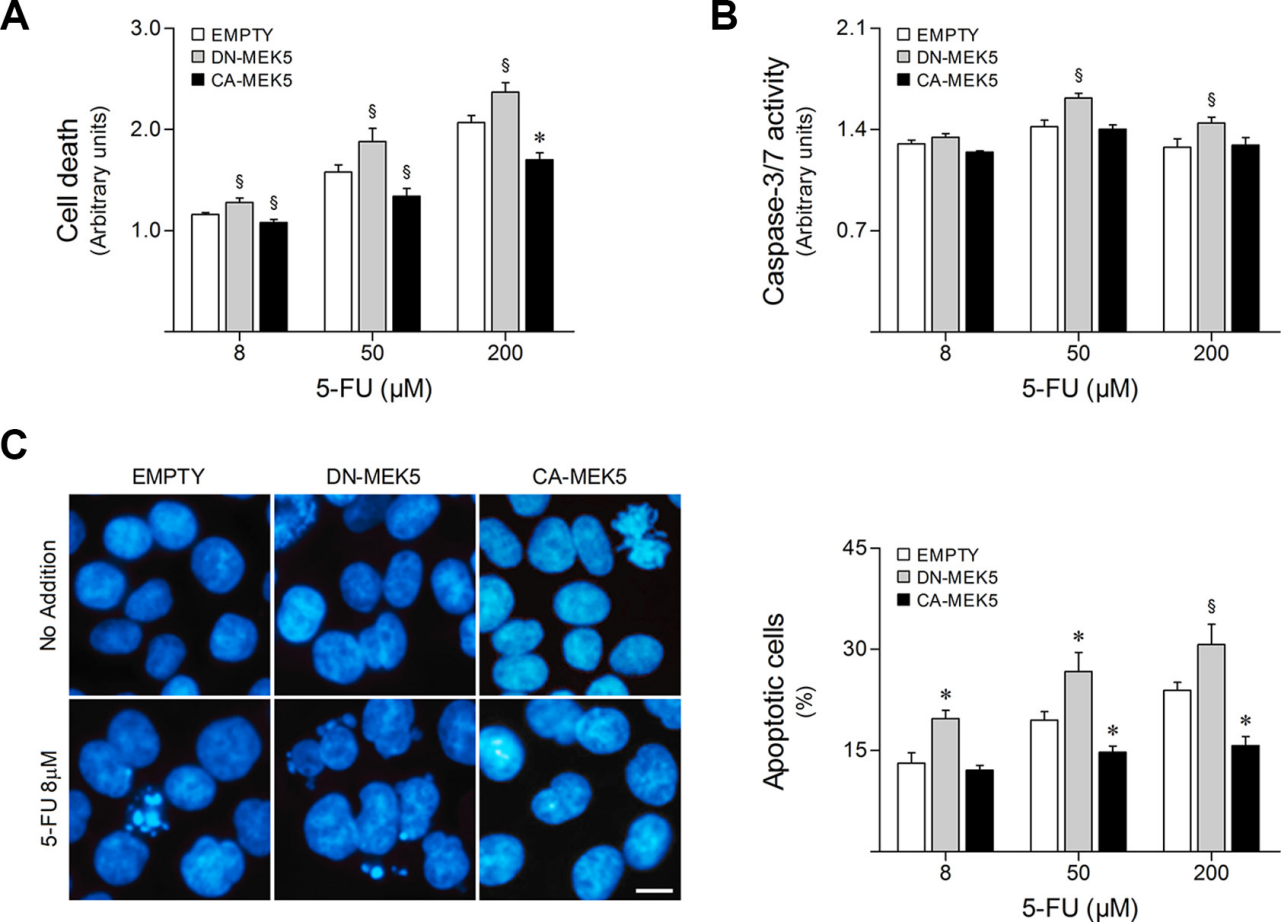
MEK5 differential activation modulates HCT116 cell sensitivity to 5-FU HCT116 cells stably expressing CA-MEK5 or DN-MEK5, and empty control, were exposed to 8, 50 and 200 μM 5-FU. DMSO was used as vehicle control. (**A**) At 48 h following treatment, general cell death was evaluated according to LDH release. (**B**) Caspase-3/7 activity was determined at 16 h after 5-FU treatment. (**C**) Nuclear morphology after Hoechst staining was evaluated by fluorescence microscopy at 24 h following treatment. Representative images of Hoechst staining at 400× magnification are shown. Scale bar, 10 μm. Results are expressed as mean ± SEM fold-change to respective vehicle control cells (A and B), or as percentage of apoptotic cells per field ± SEM (C), from at least 3 independent experiments. ^§^*p <* 0.05 and *p < 0.01 from EMPTY cells.

Having shown that 5-FU may require MEK5/ERK5 signaling inhibition to effectively trigger its anticancer effects, we next investigated whether MEK5/ERK5 differential activation could determine colon cancer cell sensitivity to this chemotherapeutic drug. For this purpose, stably transduced HCT116 cells overexpressing CA-MEK5 or DN-MEK5 were exposed to 8–200 μM 5-FU for 48 h. Cell viability and cell death were evaluated by MTS/PrestoBlue metabolism and LDH release assays, respectively. Interestingly, we found that ERK5 overactivation by CA-MEK5 increases resistance to 5-FU. In fact, CA-MEK5 expression significantly decreased cell death (Figure 3A) and increased cell viability following 5-FU treatment (Supplementary Figure S1A), compared to empty vector cells (*p* < 0.05). On the other hand, inhibition of ERK5 by DN-MEK5 enhanced 5-FU cytotoxicity, increasing general cell death after 5-FU exposure (*p* < 0.05).

5-FU is known to effectively trigger apoptotic cell death in HCT116 cells [25]. Therefore, caspase-3/7-like activity was measured after treatment with 5-FU for 16 h. Additionally, changes in nuclear morphology were assessed by fluorescence microscopy of Hoechst stained nuclei at 24 h following 5-FU treatment to detect apoptotic events. In line with cytotoxicity assay results, 5-FU-induced apoptosis was enhanced by DN-MEK5 expression, resulting in significantly increased caspase-3 and -7 activation (*p* < 0.05) (Figure 3B) as well as nuclear fragmentation (*p* < 0.05) (Figure 3C), whereas CA-MEK5 expression significantly abrogated apoptosis following 5-FU treatment, compared to empty vector cells (*p* < 0.01).

**Figure 4:**
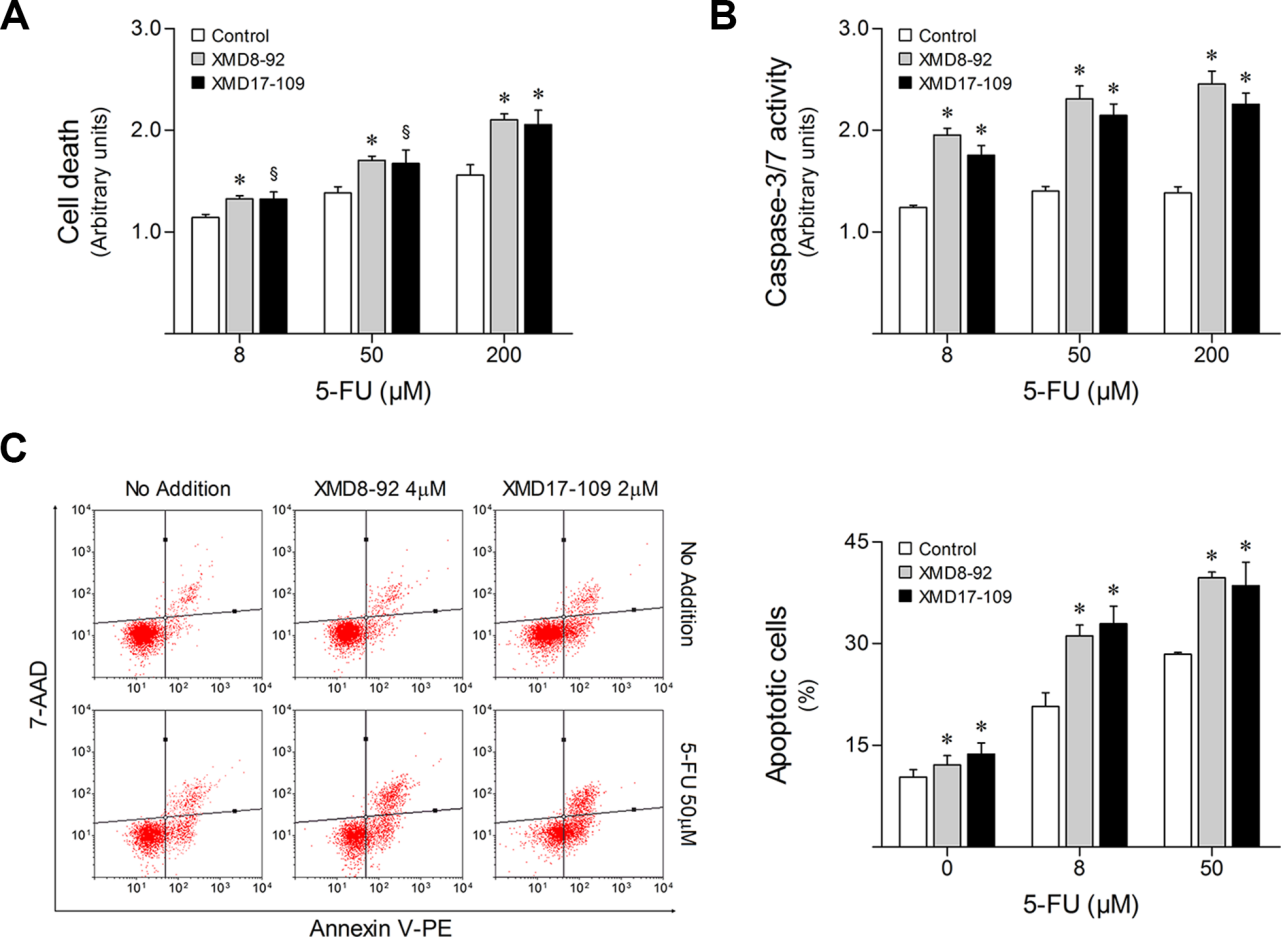
ERK5 pharmacological inhibition increases HCT116 cell sensitivity to 5-FU HCT116 cells were incubated with either 4 μM XMD8-92 or 2 μM XMD17-109, alone or in combination with 8, 50 and 200 μM 5-FU. DMSO was used as vehicle control. (**A**) At 48 h following treatment, general cell death was evaluated according to LDH release. (**B**) Caspase-3/7 activity was determined at 16 h after 5-FU treatment. (**C**) The percentage of apoptotic cells was determined by Annexin V/7-AAD (Guava Nexin assay) staining at 48 h following treatment. Representative flow cytometry plots of cells stained for Annexin V and 7-AAD are shown. Results are expressed as mean ± SEM fold-change to vehicle control cells (A and B), or as percentage of apoptotic cells ± SEM (C), from at least 3 independent experiments. ^§^*p <* 0.05 and *p < 0.01 from 5-FU single treatment.

In parallel, HCT116 parental cells were treated for up to 48 h with increasing doses of 5-FU in combination with 4 μM XMD8-92 or 2 μM XMD17-109, two highly-selective ERK5 pharmacological inhibitors (Supplementary Figure S1B) [26, 27]. In agreement with the effect of DN-MEK5, ERK5 inhibition using XMD8-92 or XMD17-109 enhanced HCT116 response to 5-FU, increasing general cell death (*p* < 0.05) (Figure 4A), while decreasing cell viability (*p* < 0.05) (Supplementary Figure S1C), compared to 5-FU single treatment. Confirming these observations, compared to 5-FU alone, the combination of 5-FU with XMD8-92 or XMD17-109 was associated with increased caspase-3/7 activity (*p* < 0.01) (Figure 4B) and percentage of apoptotic cell death as determined by flow cytometry analysis of Annexin V/7-AAD staining (*p* < 0.01) (Figure 4C).

In addition to apoptosis, autophagy has also been proposed as a major determinant of colon cancer cell response to 5-FU in HCT116 cells [28]. However, our results failed to show any evident effect of ERK5 inhibition by XMD8-92 on the LC3-II/I ratio or autophagic degradation of p62 (Supplementary Figure S2), suggesting that autophagy does not play a major role in the cellular response to 5-FU following ERK5 inhibition.

Finally, we evaluated whether the inhibition of ERK5 could also affect the sensitivity of colon cancer cells to irinotecan and oxaliplatin, two cytotoxic agents currently used in 5-FU-based chemotherapy [1, 2]. Interestingly, our results demonstrated that XMD8-92 treatment sensitizes HCT116 cells to irinotecan (*p* < 0.05) (Supplementary Figure S3), whereas no significant relationship was found between ERK5 inhibition and oxaliplatin sensitivity (data not shown).

Taken together, the aforementioned data support a major role for MEK5/ERK5 signaling in chemoresistance, particularly suggesting that ERK5 inhibition increases 5-FU apoptotic activity in colon cancer cells. Notably, the sensitizing effects of ERK5-signaling inhibition were consistently demonstrated using 8 μM 5-FU, a clinically achievable 5-FU concentration [29], emphasizing the clinical relevance of our findings.

### ERK5 inhibition increases 5-FU-induced cytotoxicity through a p53-dependent mechanism

**Figure 5:**
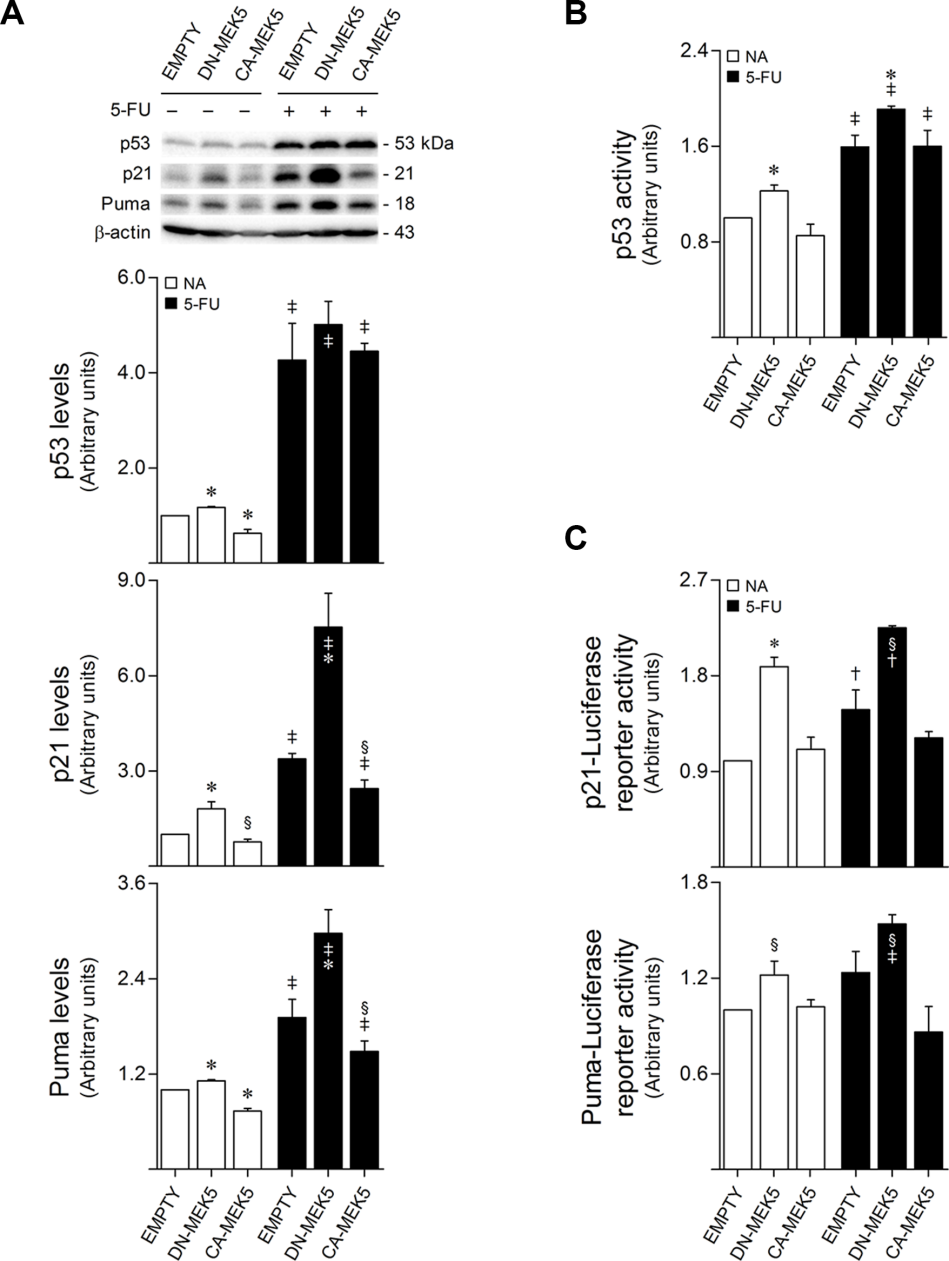
MEK5 differential activation regulates p53 transcriptional activation in HCT116 cells Stably transduced HCT116 cells overexpressing CA-MEK5 or DN-MEK5, and empty control cells, were exposed to 8 μM 5-FU. DMSO was used as vehicle control. (**A**) At 24 h after treatment, cells were harvested for total protein extraction. Protein expression levels were evaluated by western blot. Representative blots are shown. (**B**) The DNA-binding capacity of nuclear p53 was measured 24 h following treatment using the TransAM p53 assay. (**C**) p53-dependent transactivation of *p21/WAF1* (p21-Luc, upper panel) or *PUMA* (PUMA-Luc, lower panel) promoter-reporter constructs was determined at 48 h following treatment. Results are expressed as mean ± SEM fold-change from vehicle control EMPTY cells of at least 3 independent experiments. ^§^*p <* 0.05 and *p < 0.01 from EMPTY cells; ^†^*p <* 0.05 and ^‡^*p <* 0.01 from respective vehicle control cells.

The antitumor activity of 5-FU has been greatly attributed to its ability to induce both p53 accumulation and activity [17, 18], leading to the direct transcription of cell-cycle inhibitory genes, such as *p21/WAF1*, and to the upregulation of several pro-apoptotic targets, including *PUMA* [18, 30]. To elucidate the role of MEK5/ERK5 signaling in 5-FU resistance, we next investigated the relationship between MEK5/ERK5 activity and p53 expression and function in the context of 5-FU apoptotic response. Immunoblot analysis demonstrated that MEK5/ERK5 signaling blockage by DN-MEK5 significantly increased basal steady-state protein levels of p53 (*p* < 0.01), along with the expression of its transcriptional targets p21 (*p* < 0.01) and Puma (*p* < 0.01), compared to empty control cells (Figure 5A). In addition, following 5-FU treatment for 24 h, DN-MEK5 markedly increased the levels of both p21 and Puma, compared to empty cells (*p* < 0.01) (Figure 5A), although without differentially affecting 5-FU-induced p53 steady-state levels. In turn, our results showed that steady-state protein levels of p53 transcriptional targets were antagonized by MEK5 constitutive activation (*p* < 0.05) (Figure 5A). Interestingly, these results suggested that ERK5 signaling could be inhibiting p53 transcriptional activity, rather than p53 protein expression or stability.

To assess whether MEK5/ERK5 signaling was indeed modulating p53 transactivity, the DNA-binding capacity of p53 was measured in nuclear protein extracts. Remarkably, we found that ERK5 inhibition by DN-MEK5 significantly increased both basal (*p* < 0.01) and 5-FU-induced (*p* < 0.01) p53 transcriptional activation, compared with empty controls (Figure 5B). Further, using p53 transcriptional activity reporter plasmids, we confirmed that DN-MEK5 was an effective inducer of p53-mediated *p21/WAF1* and *PUMA* gene activation (*p* < 0.05), under basal conditions, and following 5-FU exposure (Figure 5C).

In agreement with these results, treatment with XMD8-92 in combination with 5-FU led to significantly increased p21 (*p* < 0.01) and Puma (*p* < 0.01) protein steady-state levels, as compared to cells treated with 5-FU alone (Supplementary Figure S4A), however without affecting p53 DNA-binding activity (Supplementary Figure S4B). Nevertheless, when coupled with 5-FU, treatment with XMD8-92 significantly increased the transcriptional activation of both *p21/WAF1* (*p* < 0.01) and *PUMA* (*p* < 0.01), compared to 5-FU single treatment (Supplementary Figure S4C), also suggesting increased p53 activity in this setting. Collectively, these results suggest a synergic relationship between ERK5 inhibition and 5-FU-triggered p53 transcriptional activation in HCT116 colon cancer cells.

**Figure 6:**
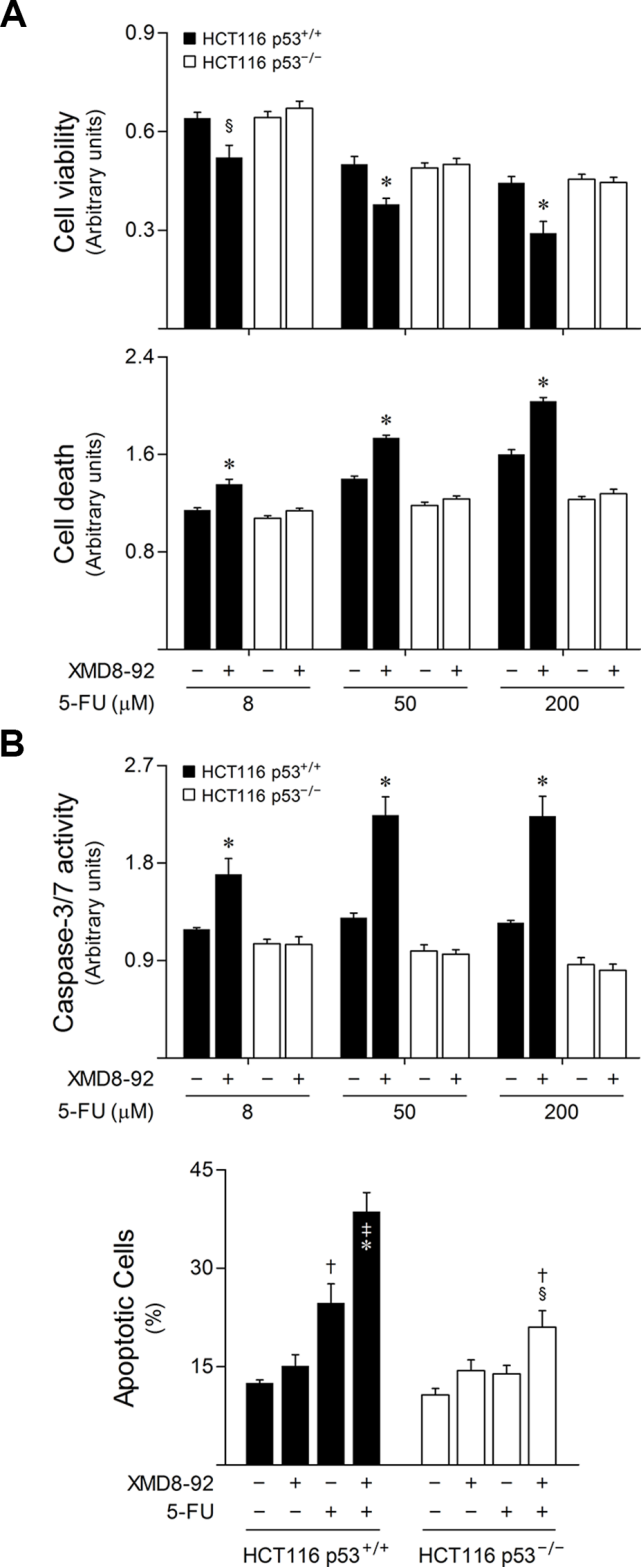
ERK5 inhibition increases 5-FU-induced apoptosis in a p53-dependent manner HCT116 p53 wild-type (p53^+/+^) and null (p53^−/−^) isogenic cell lines cells were incubated with either 8, 50 or 200 μM 5-FU, alone or together with 4 μM XMD8-92. DMSO was used as vehicle control. (**A**) At 48 h following treatment, cell viability (upper panel) and general cell death (lower panel) were evaluated by PrestoBlue metabolism and LDH release assays, respectively. (**B**) Caspase-3/7 activity (upper panel) was determined at 16 h after 5-FU treatment. The percentage of apoptotic cells (lower panel) was determined by Annexin V/7-AAD staining, 24 h following treatment using 50 μM 5-FU. Results are expressed as mean ± SEM fold-change to vehicle control cells (A and B, upper panel), or as percentage of apoptotic cells per field ± SEM, (B, lower panel) from at least 3 independent experiments. ^§^*p <* 0.05 and *p < 0.01 from 5-FU single treatment; ^†^*p <* 0.05 and ^‡^*p <* 0.01 from vehicle control cells.

In light of these findings, and to fully characterize the relevance of p53 function for the combined effects arising from 5-FU treatment and ERK5 inhibition, HCT116 p53 wild-type (p53^+/+^) and null (p53^−/−^) isogenic cell lines were exposed to 5-FU alone, or in combination with XMD8-92. Interestingly, ERK5 pharmacological inhibition markedly increased the response of HCT116 cells carrying wild-type p53 to 5-FU, as evidenced by a significant decrease in cell viability (*p* < 0.05), and an increase in general cell death (*p* < 0.01) (Figure 6A), caspase-3/7 activity and apoptotic Annexin V/7-AAD-positive cells (*p* < 0.01) (Figure 6B), compared to 5-FU single treatment. In contrast, XMD8-92 had marginal effects in sensitizing p53 null HCT116 cells to the cytotoxic effects of 5-FU. Taken together, these results indicate that ERK5 inhibition sensitizes colon cancer cells to 5-FU, at least in part, by inducing p53-dependent apoptosis, providing a functional mechanism connecting the MEK5/ERK5 cascade with 5-FU anticancer activity.

### ERK5 inhibition decreases IκB phosphorylation

**Figure 7:**
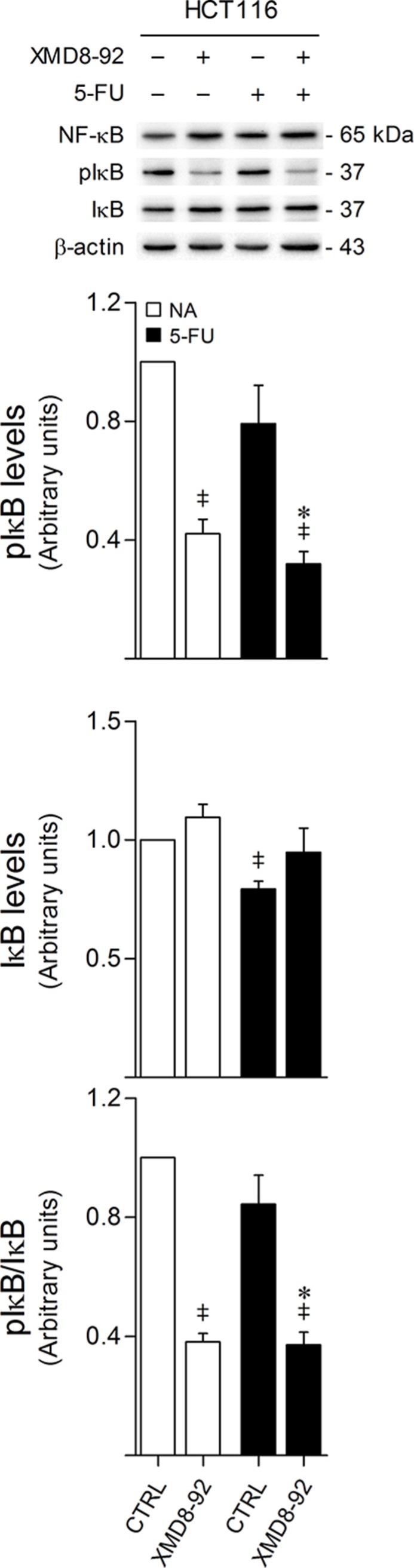
ERK5 inhibition decreases IκB phosphorylation HCT116 cells were incubated with either 8 μM 5-FU, 4 μM XMD8-92, or both. DMSO was used as vehicle control. At 24 h after treatment, cells were harvested for total protein extraction. Protein expression levels were evaluated by western blot. Representative blots are shown. Results are expressed as mean ± SEM fold-change from vehicle control cells of at least 3 independent experiments. *p < 0.01 from 5-FU single treatment; ^‡^*p <* 0.01 from vehicle control cells.

We have previously shown that ERK5 activation promotes IκB phosphorylation, targeting IκB for degradation, and leading to increased NF-κB nuclear translocation and transcriptional activity, establishing a novel axis by which MEK5/ERK5 signaling contributes to colon cancer onset, progression and metastasis [11]. Here, we investigated the relevance of the interplay between ERK5 and NF-κB signaling pathways within the context of 5-FU resistance. In line with our previous results, we found that ERK5 inhibition by XMD8-92 decreases the levels of phosphorylated IκB, either alone or in combination with 5-FU (*p* < 0.01) (Figure 7). However, no significant differences were found in NF-κB steady-state levels when treating HCT116 cells with XMD8-92 or 5-FU, precluding the hypothesized contribution of the MEK5/ERK5/NF-κB axis for 5-FU resistance.

### ERK5 inhibition enhances 5-FU antitumor activity *in vivo*

**Figure 8:**
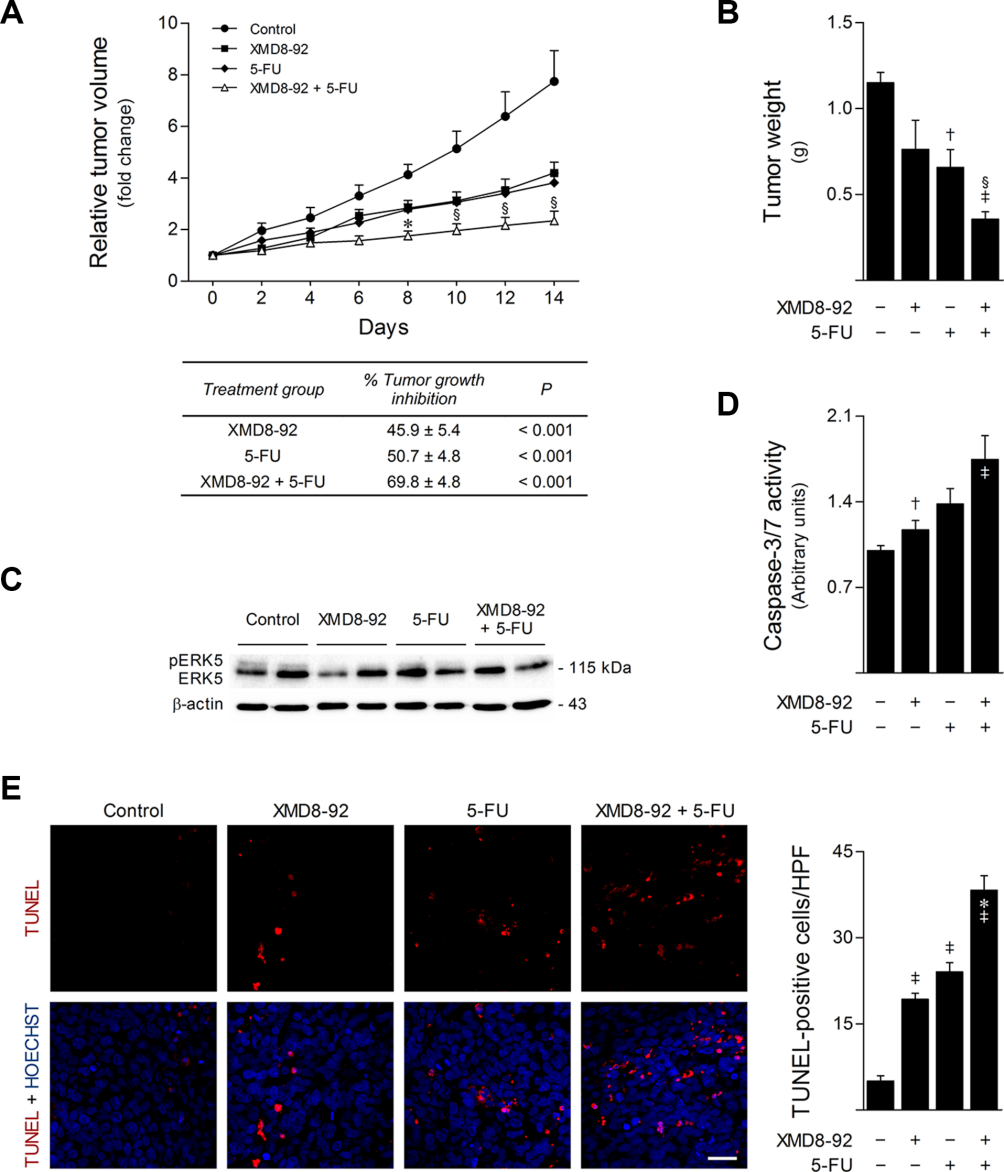
ERK5 inhibition by XMD8-92 increases *in vivo* 5-FU anticancer activity BALB/c *scid* mice (6- to 8-week-old) were subcutaneously injected with 1.5 × 10^6^ HCT116 cells. When tumor volume reached ~150 mm^3^, mice were intraperitoneally injected every two days with either vehicle control, 40 μg/g 5-FU, 50 μg/g XMD8-92, or combination therapy for 14 days (*n* = 6 per group). (**A**) Tumor volume growth curve (top). Results are expressed as mean ± SEM fold-change to treatment start. Tumor growth inhibition compared with control mice was calculated at the last day of treatment (bottom). (**B**) Final tumor weight. (**C**) ERK5 protein levels in total protein lysates from snap frozen tumor xenograft samples were evaluated by western blot. Representative blots are shown. (**D**) Caspase-3/7 activity was evaluated in tumor protein extracts using the Caspase-Glo 3/7 assay. Results are expressed as mean ± SEM fold-change to vehicle treated tumors. (**E**) Representative microphotographs of TUNEL analysis (400× magnification), with apoptotic cells (red) and Hoechst stained-nuclei (blue) (left panel), and respective quantification of TUNEL-positive cells per high-power field (HPF) (right panel). Scale bar, 30 μm. Results are expressed as mean ± SEM. ^§^*p <* 0.05 and *p < 0.01 from 5-FU single treatment; ^†^*p <* 0.05 and ^‡^*p <* 0.01 from vehicle control treatment.

To clearly establish the role of MEK5/ERK5 signaling in colon cancer response to 5-FU *in vivo*, we next evaluated the effect of XMD8-92 and 5-FU combinatorial treatment in a murine tumor xenograft model. HCT116 cell suspensions were injected subcutaneously into the dorsal interscapular region of BALB/c *scid* mice. When tumors reached ~150 mm^3^, mice were randomized into four treatment groups (*n* = 6 per group): control, XMD8-92, 5-FU, and XMD8-92 + 5-FU. After 14 days of treatment, XMD8-92 and 5-FU monotherapy inhibited tumor growth by 46 and 51%, respectively, compared to vehicle control-treated mice (*p* < 0.001). However, when XMD8-92 was administered in combination with 5-FU, tumor growth inhibition was improved to 70% (*p* < 0.001) (Figure 8A). Of note, this combinatorial treatment was also more efficient than 5-FU alone (*p* < 0.05), recapitulating our *in vitro* results. Similarly, final tumor weights were significantly reduced upon combination of XMD8-92 with 5-FU, as compared with 5-FU monotherapy (*p* < 0.05) and vehicle control-treated tumors (*p* < 0.01) (Figure 8B). Importantly, analysis of ERK5 steady-state levels in tumor xenograft samples validated the inhibition of ERK5 phosphorylation by XMD8-92 *in vivo* (Figure 8C). Finally, apoptosis was evaluated according to caspase activity in tumor protein extracts and TUNEL analysis in histological sections. Consistent with our *in vitro* observations, caspase-3/7 activities were increased in tumors from treated animals (*p* < 0.05) (Figure 8D). Moreover, this was further associated with an increase of nearly 40% of TUNEL-positive cells in tumor sections from mice receiving XMD8-92 and 5-FU combination treatment, as compared to 5-FU monotherapy (*p* < 0.01) or vehicle-treated tumors (*p* < 0.01) (Figure 8E). Altogether, these results demonstrate that pharmacological inhibition of ERK5 using XMD8-92 strongly enhances the anticancer activity of 5-FU *in vivo*, emphasizing the importance of the MEK5/ERK5 signaling cascade in 5-FU resistance.

## DISCUSSION

In colon cancer, there is clinical evidence supporting that MEK5/ERK5 signaling dysregulation plays an important part in tumorigenesis [11, 12]. In this regard, we have recently demonstrated that MEK5 and ERK5 are overexpressed in human colon adenomas and adenocarcinomas, suggesting that MEK5/ERK5 signaling overactivation may be involved in tumor initiation and progression [11]. In the present study, we showed that increased ERK5 expression is associated with shorter overall survival in colon cancer patients, providing further insight into the clinical relevance of the MEK5/ERK5 signaling pathway, and adding to the current state-of-the-art, where increased phosphorylated MEK5 in colon tumors has been associated with poorer 5-year disease-free and overall survival rates [12]. Additionally, ERK5 overexpression has been identified as an independent prognostic marker in both breast [31] and prostate cancer [32].

Although the full extent of MEK5/ERK5 signaling contribution to cancer remains incompletely understood, a central role in sustaining proliferation is currently recognized [13, 14]. Indeed, previous studies showed that activated ERK5 may support proliferation by promoting G1-S [33] and G2-M phase transitions [34]. Further, several mitogens and oncogenic signals are known to transduce their pro-growth signaling through the MEK5/ERK5 pathway [35]. In this study, to elucidate the function of MEK5/ERK5 signaling cascade in colon cancer pathogenesis, we have shown that MEK5 constitutive activation induces proliferation in two well-established cellular models of colon cancer. Importantly, these observations are consistent with our previous report demonstrating that ERK5 signaling upregulation in colon cancer cells accelerates cell cycle progression by activating NF-κB [11], and reinforce the relevance of this unique cascade in promoting abnormal growth.

Several lines of evidence have been accumulating to support the crucial contribution of the MEK5/ERK5 pathway as a mediator of therapy resistance. Remarkably, it was recently reported that MEK5 and ERK5 mRNA overexpression associates with poor survival in breast cancer patients after chemotherapy, whereas patients with low expression of these kinases have been shown to benefit from systemic treatment [36]. In line with these observations, ERK5-signaling inhibition has been found to sensitize tumor cells to several antitumor agents, including etoposide [37], trastuzumab [31], tamoxifen [38], crizotinib [39], doxorubicin [40–42], docetaxel [41] and cisplatin [40, 43]. In this study, using complementary *in vitro* and *in vivo* colon cancer models, we show for the first time that the MEK5/ERK5 pathway is an important modulator of 5-FU anticancer properties, the cornerstone agent in colon cancer treatment, further expanding the relevance of MEK5/ERK5 signaling in chemoresistance. In this regard, we have shown that KRAS/MEK5/ERK5 protein expression and activation levels are impaired following 5-FU treatment, both in HCT116 and SW620 cells, strongly suggesting that 5-FU may require the silencing of this signaling pathway to effectively trigger its cytotoxic effects. In agreement with this hypothesis, MEK5 constitutive activation prevented the pro-apoptotic activity of 5-FU in HCT116 cells. Conversely, using complementary genetic and pharmacological approaches, we report that ERK5 inhibition sensitized HCT116 cells to 5-FU-induced apoptosis, providing novel clues for a link between MEK5/ERK5 signaling and tumor cell response to 5-FU. Finally, our findings were further validated *in vivo* in a HCT116 xenograft model. In this context, combination therapy using the ERK5 inhibitor XMD8-92 together with 5-FU was shown to significantly enhance apoptosis and impair tumor growth, compared to either treatment alone, attesting to the potential of this dual therapeutic approach in a preclinical setting. Taken together, these results provide compelling evidence supporting the rationale for ERK5 signaling inhibition, combined with current 5-FU-based chemotherapy, as a promising new therapeutic approach for colon cancer treatment.

Regarding the mechanism by which MEK5/ERK5 signaling inhibition enhances colon cancer cell sensitivity to 5-FU, our results indicate that they are largely dependent on p53. In agreement with our results, ERK5 has already been reported as a negative regulator of p53. Particularly, activated ERK5 was shown to induce CHIP ubiquitin ligase activity, consequently promoting CHIP-mediated ubiquitination and degradation of p53 [22]. In addition, activated ERK5 associates with PML, disrupting PML-dependent MDM2 nucleolar sequestration, once again facilitating p53 ubiquitination and subsequent degradation [23]. However, whereas these studies have reasoned that ERK5 activity compromises p53 protein stability [22, 23], our data rather suggests that ERK5 antagonizes p53 transcriptional activity, with only small effects in p53 protein levels. In fact, we demonstrate that inhibition of ERK5 increases p53-dependent transcriptional activation of p21 and Puma, suggesting a potential axis by which ERK5 inhibition might be enhancing apoptosis when coupled with 5-FU treatment. Strengthening our case, genetic and pharmacological strategies employed to inhibit ERK5 signaling showed, for the most part, overlapping cellular and molecular effects. However, the differences between these two approaches need to be taken into account, as they may explain why XMD8-92 was not sufficient to induce significant changes in p53 DNA-binding activity. In this respect, while genetically blocking MEK5 activity is expected to specifically target ERK5, XMD8-92 may display off-target effects, as already reported in pancreatic tumor cells at higher concentrations [44]. With this in mind, one may hypothesize that unknown nonspecific targets of XMD8-92, other than ERK5, could be partially counterbalancing the impact of ERK5 pharmacological inhibition. Future studies will be necessary to fully determine the routes leading to p53 activation upon ERK5 inhibition, since patients retaining wild-type *P53* might particularly benefit from the combination of anti-ERK5 therapies to improve the efficacy of standard-of-care chemotherapy.

In a broader point of view, several other members of the MAPK family have been proposed to regulate tumor cell response to 5-FU, specifically in colon cancer. For instance, inhibition of MEK1/2 enhanced the radiosensitizing effects mediated by 5-FU *in vitro* and *in vivo* [45]. Further, JNK activity has been reported to prompt autophagy, protecting colon cancer cells with impaired p53 function from 5-FU cytotoxic effects, which in turn was shown to be reversed following JNK inhibition [46]. Instead, the p38MAPK pathway was shown to be activated in response to 5-FU, controlling cell fate by shifting the balance between apoptosis and autophagy towards increased 5-FU sensitivity [47]. Remarkably, the effects of p38MAPK and JNK in controlling 5-FU response have also been shown to be p53-dependent [46, 47]. However, unlike p38MAPK and JNK, the role of ERK5 in the cellular response to 5-FU appears to be independent of any influence on the autophagic pathways. Nevertheless, further investigations are needed to evaluate the effects of a possible crosstalk between MEK5/ERK5 signaling and the remaining MAPK family members.

In conclusion, the present study demonstrates that MEK5/ERK5 overactivation contributes to colon cancer aggressiveness and therapy resistance, underlining the relevance of this cascade to the hallmarks of colon cancer. Further, our results reveal that ERK5 signaling inhibition enhances the anticancer properties of 5-FU, at least in part by promoting p53-dependent apoptosis. In this framework, MEK5 and ERK5 emerge as attractive targets for colon cancer treatment and chemosensitization. To date, several small-molecule inhibitors have been designed to specifically target the MEK5/ERK5 pathway [26, 27, 48]. Equally important, TG02, a new oral pyrimidine-based multikinase inhibitor [49, 50], is also known to directly block ERK5 activity [51]. In fact, this promising antitumor agent is currently undergoing phase I trials in leukemia and multiple myeloma patients, providing the first clues to the benefits of targeting the MEK5/ERK5 cascade in clinical practice, and encouraging other ERK5-specific inhibitors such as XMD8-92 to progress into clinical evaluation.

## MATERIALS AND METHODS

### Online database survival analysis

The prognostic value of ERK5 mRNA expression was assessed using the SurvExpress web resource [24] to establish correlations with patient outcome. Kaplan-Meier survival curves were generated using two publicly available colon cancer datasets: The Cancer Genome Atlas (TCGA) colorectal database (http://cancergenome.nih.gov/), and the colon cancer SurvExpress metabase, which combines multiple datasets from the Gene Expression Omnibus (GEO) repository (http://www.ncbi.nlm.nih.gov/geo/). ERK5 mRNA expression was queried in the records using the NCBI Entrez Gene identifier #5598. For each dataset, results were obtained using the average expression score from duplicate ERK5 probe sets and the original quantile-normalized data. Samples were split into two maximized risk groups to generate low-risk and high-risk groups. Overall survival was used as the endpoint analysis for both datasets. Data was retrieved from the SurvExpress server between June 2015 and July 2015 (http://bioinformatica.mty.itesm.mx/SurvExpress).

### Cell culture

HCT116 and SW620 human colorectal carcinoma cell lines were obtained from ECACC, (Porton Down, Wiltshire, UK). HCT116 p53 wild-type (p53^+/+^) and null (p53^−/−^) isogenic cell line were obtained from GRCF Cell Center and Biorepository (Johns Hopkins University, School of Medicine, Baltimore, MD, USA). HCT116 cell lines were grown in McCoy's 5A modified medium, and SW620 cells in Dulbecco's modified Eagle's medium (DMEM) (both from Gibco, Life Technologies, Paisley, UK). Media were supplemented with 10% heat-inactivated fetal bovine serum and 1% antibiotic/antimycotic solution (both from Gibco). Cell lines were cultured at 37°C under a humidified atmosphere of 5% CO_2_.

### Lentiviral particle production

pWPI-GFP lentiviral expression constructs encoding constitutively active (CA)-MEK5 (MEK5DD; Ser313 and Thr317 activating phosphorylation residues substituted with Asp), or dominant negative (DN)-MEK5 (MEK5AA; Ser311 and Thr315 replaced with Ala), and the corresponding empty vector control, were kindly provided by Dr. Robert C. Doebele [52]. Lentiviral particles were produced by co-transfecting HEK293T cells with the packaging plasmids pGag-pol and pRev, the envelope plasmid pVSV-G, and the lentiviral expression vector encoding either CA-MEK5, DN-MEK5 or empty control, at a 3:2:1:4 ratio. Transfections were performed using Lipofectamine 2000 (Invitrogen, Life Technologies, Paisley, UK), according to manufacturer's instructions. Lentivirus-containing supernatants were harvested 48 and 72 h after transfection, pooled and filtered through a 0.22 μm filter to remove cell debris. Harvested lentiviruses were stored at −80°C until use.

### Generation of stable colon cancer cell lines with differential MEK5 activation

To establish stable cell lines with differential MEK5 activation, HCT116 and SW620 cells were seeded in 6-well plates at a density of 3 × 10^5^ cells/well. Twenty-four hours after plating, cells were infected by adding thawed lentivirus-containing supernatants. Transduction was enhanced by spinoculation at 500 *g* for 1 h at 25°C. Lentiviral supernatants were replaced for fresh medium 12 h after transduction. As the pWPI backbone allows for the simultaneous expression of the transgene together with an eGFP marker on transduced cells, stable cell lines were purified by sorting GFP-expressing cells on a BD FACSAria I cell sorter (BD Biosciences, San Jose, CA, USA). Following cell sorting, MEK5 overexpression and ERK5 differential activation status were confirmed by immunoblotting. The percentage of GFP-positive cells was regularly monitored to assure > 80% cells expressing the transgenes in all experiments.

### Cell proliferation

*In vitro* cell growth was assessed using the CellTiter 96 AQ_ueous_ Non-Radioactive Cell Proliferation Assay (Promega, Madison, WI, USA), which is based on the bioreduction of the 3 (4, 5-dimethylthiazol-2-yl)-5-(3-carboxymethoxyphenyl)-2-(4-sulfophenyl)-2H-tetrazolium inner salt (MTS) to a water soluble formazan product. Briefly, HCT116- and SW620-derived cell lines were seeded in 96-well plates at a density of 5 × 10^3^ and 1 × 10^4^ cells/well, respectively, and MTS metabolism was assayed at 4, 24, 48 and 72 h after plating. For this purpose, 20 μL of MTS/PMS solution (19:1) was added to culture medium at the indicated time-points, and absorbance readings were measured at 490 nm, using a Model 680 microplate reader (Bio-Rad, Hercules, CA, USA).

Additionally, the distribution of cells along G0/G1, S and G2/M phases of the cell cycle was used to determine the proliferating fraction of asynchronously growing populations in culture. For this purpose, the stably transduced HCT116 and SW620 cell lines were seeded in 6-well plates at 1.5 × 10^5^ and 3 × 10^5^ cells/well, respectively. Seventy-two hours after plating, attached cells were washed with PBS, harvested, and collected by centrifugation at 800 *g* for 5 min at 4°C. Harvested cells were then resuspended in cold PBS, and fixed by drop wise addition of an equal volume of 80% ice-cold ethanol, under gentle shaking. Subsequently, cells were incubated for 30 min on ice, and stored for at least 18 h at 4°C. Before cell cycle analysis, fixed cells were resuspended in a propidium iodide solution (PBS containing 50 μg/mL RNase A and 25 μg/mL propidium iodide (both from Sigma-Aldrich, St. Louis, MO, USA)), and incubated at 37°C for 30 min, for DNA staining. DNA content was determined according to fluorescence intensity using a BD LSRFortessa Cell Analyser cytometer (BD Biosciences). Cell cycle distribution was evaluated using the ModFit LT software (version 4.0; Verity Software House, Topsham, ME, USA). Cell proliferation index (PI) was calculated using the following equation: PI = (percentage of S + G2 + M-phase cells), as previously described [53].

### Cell treatment

5-FU (Sigma-Aldrich), XMD8-92 (Tocris Bioscience, Bristol, UK) and XMD17-109 (MedChem Express, NJ, USA) stock solutions were prepared in dimethyl sulfoxide (DMSO) and stored at −80°C. Before treatments, cells were allowed to adhere for 24 h, and then incubated with either 5-FU alone or in combination with XMD8-92 or XMD17-109, at the indicated concentrations, for the indicated times. For cytotoxicity assays, HCT116 cells were seeded in 96–well plates, at 1 × 10^4^ cells/well, and next exposed to 8–200 μM 5-FU and/or 4 μM XMD8-92 or 2 μM XMD17-109 for 48 h. For the morphological detection of apoptotic nuclei, stably transduced HCT116 cells were seeded into 35 mm dishes, at a density of 1.5 × 10^5^ cells/dish, and exposed to 8–200 μM 5-FU for 24 h. For the Guava Nexin assay, HCT116 cells were seeded in 24-well plates, at 5 × 10^4^ cells/well, and exposed to 8–50 μM 5-FU and/or 4 μM XMD8-92 or 2 μM XMD17-109 for 48 h. For the evaluation of caspase-3/7 activity, HCT116 cells were seeded on a 96-well plate at 1.5 × 10^5^ cells/well, and exposed to 8–200 μM 5-FU and/or 4 μM XMD8–92 or 2 μM XMD17-109 for 16 h. All experiments were performed in parallel with DMSO vehicle control. Final DMSO concentration was always 0.1%.

### Evaluation of cell death and viability

MTS metabolism was assessed as a measure of cell viability using the CellTiter 96 AQ_ueous_ Non-Radioactive Cell Proliferation Assay (Promega), according to manufacturer's instructions. Changes in absorbance were measured at 490 nm, using a Model 680 microplate reader (Bio-Rad, Hercules, CA, USA). Alternatively, cell viability was evaluated according to razurin metabolism using the PrestoBlue Cell Viability Reagent (Invitrogen, Life Technologies), following manufacturer's instructions. Fluorescence emission was detected using a GloMax-Multi+ Detection System (Promega) with a 525 nm excitation filter and a 580–640 nm emission filter.

General cell death was evaluated using lactate dehydrogenase (LDH) Cytotoxicity Detection Kit^PLUS^ (Roche Diagnostics GmbH, Mannheim, Germany), by measuring the amount of cytosolic LDH released from plasma membrane-damaged cells into the extracellular medium. Briefly, 50 μL of culture supernatant was collected from each well and added to a new 96-well plate to evaluate LDH release. In parallel, cells on the original plate were incubated for 15 min with lysis solution diluted in 50 μL of medium, to completely lyse the remaining cells and release their intracellular LDH into medium. Subsequently, supernatant samples and total cell lysates were incubated with 50 μL of assay substrate for 10 to 30 min, at room temperature, protected from light. Absorbance readings were measured at 490 nm, with 620 nm reference wavelength, using a Model 680 microplate reader (Bio-Rad). The percentage of LDH release was determined as the ratio between the released LDH (supernatant) and the total LDH (supernatant + cell lysate), as previously described [54].

### Evaluation of apoptotic cell death

The DNA-binding stain Hoechst was used to identify apoptotic nuclei. For this purpose, attached cells were fixed with 4% paraformaldehyde in PBS for 20 min, washed with PBS, and stained with 5 μg/mL Hoechst 33258 (Sigma-Aldrich) in PBS for 15 min. Subsequently, cells were washed with PBS and mounted with coverslips using PBS/glycerol (3:1). Nuclear morphology was evaluated by fluorescence microscopy using an AxioScope. A1 microscope (Carl Zeiss Microscopy GmbH, Jena, Germany), under 400× magnification. A minimum of five random microscopic fields with approximately 100 nuclei were counted for each condition. Fluorescent nuclei were categorized according to condensation and staining characteristics of chromatin, and the results were expressed as the percentage of apoptotic nuclei per field.

Alternatively, the percentage of apoptotic cells was assessed using the Guava Nexin Assay (Merck Millipore, Darmstadt, Germany). This assay relies on Annexin V-PE to detect phosphatidyl serine translocation to the external membrane of apoptotic cells, and the cell-impermeable dye 7-AAD as an indicator of membrane structural integrity to distinguish late apoptotic and death cells. Briefly, floating and adherent cells were collected by centrifugation at 500 *g* for 5 min, resuspended in PBS/2% FBS, and then stained with an equal volume of Guava Nexin reagent for 20 min, protected from light. Sample acquisition and analysis were performed in a Guava easyCyte 5HT flow cytometer using the Nexin software module (Merck Millipore).

### Evaluation of caspase-3/7 activity

The activation of effector caspases was used as an early marker of apoptosis. Accordingly, caspase-3 and -7 activity was measured using the Caspase-Glo 3/7 Assay (Promega). This assay is based on the cleavage of a proluminescent substrate containing the specific DEVD sequence recognized by caspase-3 and -7 to release aminoluciferin in cell lysates, which can be subsequently cleaved by luciferase, generating a luminescent signal. For this purpose, 75 μL of Caspase-Glo 3/7 reagent was added to each well, and plates were mixed by orbital shaking for 30 s. Subsequently, the mixture was incubated at room temperature for 30 min. The resulting luminescence was measured using the GloMax-Multi+ Detection System (Promega). Additionally, the activity of caspases was measured in tumor protein extracts (15 μg) using the Caspase-Glo 3/7 Assay, as previously described [55].

### p53 transcriptional activity reporter assays

The DNA binding capacity of p53 was assayed using the TransAM p53 transcription factor assay kit (Active Motif, Carlsbad, CA), according to the manufacturer's protocol. This enzyme-linked immunosorbent assay (ELISA) assay uses immobilized oligonucleotides containing p53 consensus binding site to detect active p53. A total of 5 μg of each nuclear protein extract was used for this experiment. Additionally, p53 transcriptional activation was assessed based on luciferase reporter constructs harboring the *p21/WAF1* (WWP-Luc; #16451) or the*PUMA* (PUMA Frag1-Luc; #16591) promoter (Addgene, Cambridge, MA), both comprising p53 responsive elements. The empty pBV-Luc vector was used as negative control (plasmid #16539; Addgene). Renilla luciferase activity was measured for transfection efficiency normalization by co-transfecting cells with the pRL-SV40 vector (Promega). HCT116 cells were seeded at 1 × 10^5^ cells/well on 96-well plates, and co-transfected with 100 ng of luciferase reporter constructs and 10 ng of pRL-SV40 vector, using Lipofectamine 3000 (Invitrogen). Cells were treated 24 h after transfection with 8 μM 5-FU and/or 4 μM XMD8-92. Reporter assays were performed 24 to 48 h post-treatment using the Dual-Luciferase Reporter Assay System (Promega).

### Tumor xenograft mouse model

For xenograft tumor formation, a total of 1.5 × 10^6^ HCT116 cells was resuspended in PBS (50 μl) and injected subcutaneously into the dorsal interscapular region of 6- to 8-week-old BALB/*c scid* mice. When tumors reached ~150 mm^3^, mice were randomized into four groups (*n* = 6 per group): control, XMD8-92, 5-FU, and XMD8-92 + 5-FU. Mice were injected intraperitoneally every two days with either vehicle control (30% hydroxypropyl-β-cyclodextrin), 5-FU (40 μg/g) and/or XMD8-92 (50 μg/g). Tumor size was regularly measured with a caliper, and volumes determined according to V (mm^3^) = 0.52 × L × W^2^, where L and W represent the longest and shortest axis of the tumor, respectively. Relative tumor volumes (RTV) were determined for each animal as the ratio between volumes at the indicated day and volumes at the start of treatment. The percentage of tumor growth inhibition (% TGI) was then calculated according to % TGI = 100 − (T/C × 100), where T represents the mean RTV of the treated groups, and C the mean RTV of the vehicle control group, at the last day of treatment. Mice were sacrificed with CO_2_ narcosis 14 days after treatment start. At excision, tumors were weighed and then sectioned into two equal portions; one half was fixed in 4% paraformaldehyde, and routine-processed for paraffin-embedding; the other half was rinsed with PBS, snap frozen in liquid nitrogen and stored at −80°C for subsequent protein extraction. All animal-handling procedures were performed according to EU recommendations for good practices and animal welfare, and approved by the IMM Animal Care and Ethical Committee (AEC_2014_08_PB_Cancer).

### TUNEL assay

For *in situ* detection and quantitation of apoptosis, DNA fragmentation was detected by the terminal deoxynucleotidyl transferase dUTP nick end labeling (TUNEL) assay, using the ApopTag Red *In Situ* Apoptosis Detection Kit (Merck Millipore). Tumor specimens were then counterstained with 5 μg/mL Hoechst 33258 (Sigma-Aldrich). TUNEL-positive cells were detected by fluorescence microscopy using an AxioScope.A1 microscope (Carl Zeiss Microscopy GmbH), under 400× magnification. Apoptotic frequency was quantified in tumor sections displaying similar cell density, avoiding necrotic areas, and results expressed as the mean number of TUNEL-positive cells per field.

### Total protein isolation and immunobloting

Total protein extracts were prepared from tumor cell cultures and xenograft tissues. For this purpose, samples were homogenized in lysis buffer containing 10 mM Tris-HCl (pH 7.6), 2.5 mM MgCl_2_, 0.75 mM KAc, 0.5% Nonidet P-40, 1 mM dithiothreitol (DTT) and 1× Halt Protease and Phosphatase Inhibitor Cocktail (EDTA-free; Pierce, Thermo Fisher Scientific, Rockford, IL, USA) for 30 min. Protein lysates were sonicated and centrifuged at 10000 *g*, for 10 min at 4°C. Total protein extracts were stored at −80°C. Protein concentrations were determined using the Bio-Rad Protein Assay reagent, according to the manufacturer's instructions. Steady-state protein expression levels were determined by immunoblot analysis. Briefly, 40–80 μg of total protein extracts were denatured, separated on 8% or 12% sodium dodecyl sulphatepolyacrylamide electrophoresis gels, and transferred onto nitrocellulose membranes. After blocking with 5% milk solution, blots were incubated overnight at 4°C with primary rabbit antibodies reactive to ERK5 (#3372; Cell Signaling Technology Inc., Beverly, MA, USA), LC3 (#PA1-16931; Thermo Fisher Scientific), p-MEK5 (#sc-135702), p21 (#sc-397), NF-κB (#sc-372) or IκB-α (#sc-371; all from Santa Cruz Biotechnology Inc.); with primary mouse antibodies against MEK5 (#sc-135986), KRAS (#sc-30), p53 (#sc-126; all from Santa Cruz Biotechnology Inc.), p-IκB-α (#9246; Cell Signaling Technology Inc.), or p62 (#ab56416; Abcam plc, Cambridge, UK); or with a primary goat antibody reactive to Pumaα (#sc-20534; Santa Cruz Biotechnology Inc.). Next, membranes were incubated with horseradish peroxidase-conjugated anti-rabbit, -mouse or -goat immunoglobulin secondary antibodies (Bio-Rad), for 3 h at room temperature. Finally, the proteins of interest were detected by chemiluminescence with SuperSignal reagents (Pierce, Thermo Fisher Scientific), and acquired using the ChemiDoc XRS^+^ imaging system (Bio-Rad). β-actin (#A5541; Sigma-Aldrich) was used as loading control.

### Statistical analysis

All data are expressed as mean ± standard error of mean (SEM) from at least three independent experiments. For *in vitro* and *in vivo* assays, statistical significances were determined using two-tailed Student's *t*-test. For Kaplan-Meier survival analyses a log-rank test was performed. Values of *p* < 0.05 were considered statistically significant.

## SUPPLEMENTARY MATERIALS FIGURES


