# Supplementary material for: MEK5/ERK5 signaling inhibition increases colon cancer cell sensitivity to 5-fluorouracil through a p53-dependent mechanism
Source: Oncotarget. 2016 Apr 29;7(23):34322–40. doi: 10.18632/oncotarget.9107 (PMC5085159; doi:10.18632/oncotarget.9107)
Supplement: Supplementary file 1 [file oncotarget-07-34322-s001.pdf]

MEK5/ERK5 signaling inhibition increases colon cancer cell sensitivity to 5-fluorouracil through a p53dependent mechanism

Supplementary Materials

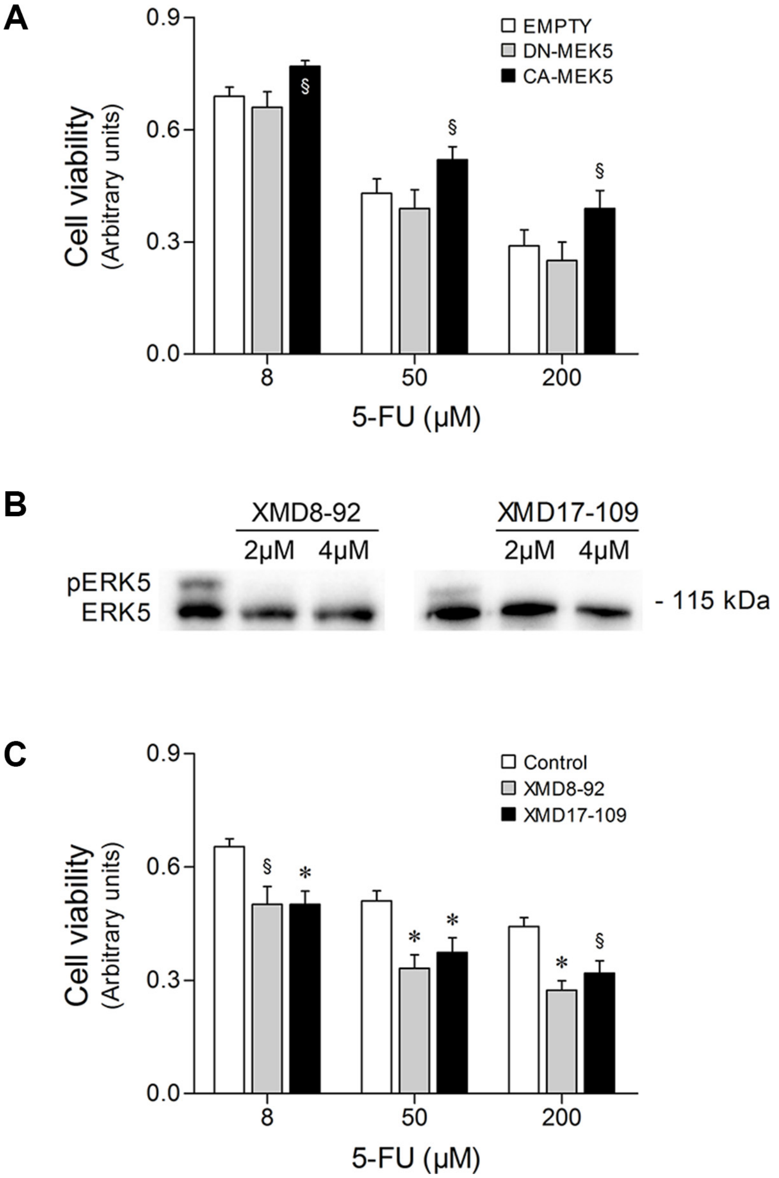

Supplementary Figure S1: MEK5/ERK5 differential activation modulates HCT116 cell viability following 5-FU treatment. (**A**) HCT116 cells stably expressing CA-MEK5 or DN-MEK5, and empty control, were exposed to 8, 50 and 200 µM 5-FU. DMSO was used as vehicle control. At 48 h following treatment, cell viability was evaluated according to MTS metabolism. Alternatively, parental HCT116 cells were incubated with either 4 µM XMD8-92 or 2 µM XMD17-109, alone or in combination with 8, 50 and 200 µM 5-FU. (**B**) Representative immunoblots of XMD8-92 and XMD17-109 effect on ERK5 phosphorylation are shown. (**C**) Cell viability was assessed according to PrestoBlue metabolism 48 h following treatment. Results are expressed as mean ± SEM fold-change to respective vehicle control cells, from at least 3 independent experiments. $^{§}p < 0.05$ and $^{*}p < 0.01$ from EMPTY cells (A) or 5-FU single treatment (C).

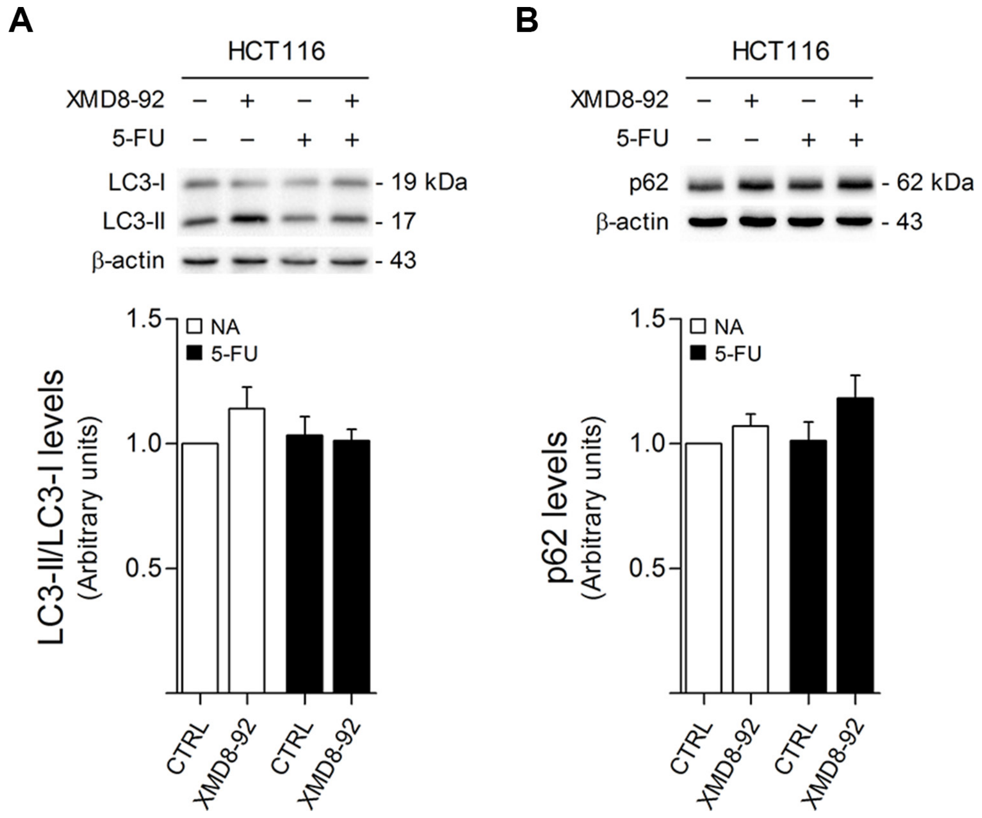

Supplementary Figure S2: Increased response to 5-FU upon ERK5 inhibition does not appear to be dependent on autophagy. HCT116 cells were incubated with either 8 µM 5-FU, 4 µM XMD8-92, or both. DMSO was used as vehicle control. At 24 h after treatment, cells were harvested for total protein extraction. LC3-II/I ratio (**A**) or autophagic degradation of p62 (**B**) were evaluated by western blot. Representative blots are shown. Results are expressed as mean ± SEM fold-change from vehicle control cells of at least 3 independent experiments.

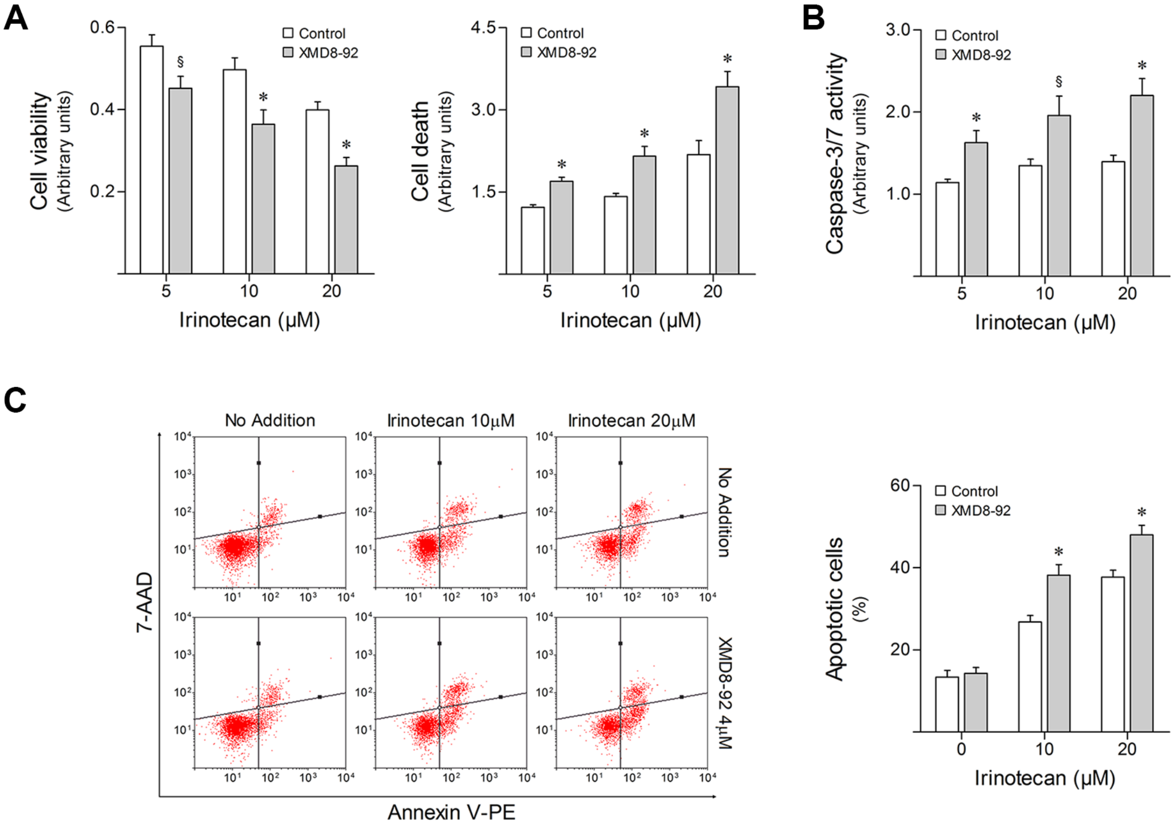

Supplementary Figure S3: ERK5 inhibition with XMD8-92 increases HCT116 cell sensitivity to irinotecan. HCT116 cells were incubated with either 5, 10 or 20 µM irinotecan, 4 µM XMD8-92, or both. DMSO was used as vehicle control. (**A**) At 48 h following treatment, cell viability (left panel) and general cell death (right panel) were evaluated by PrestoBlue metabolism and LDH release assays, respectively. (**B**) Caspase-3/7 activity was determined at 16 h after irinotecan treatment. (**C**) The percentage of apoptotic cells was determined by Annexin V/7-AAD (Guava Nexin assay) staining at 48 h following treatment. Representative flow cytometry plots of cells stained for Annexin V and 7-AAD are shown. Results are expressed as mean ± SEM fold-change to vehicle control cells (A and B), or as percentage of apoptotic cells ± SEM (C), from at least 3 independent experiments. $^{§}p < 0.05$ and $^{*}p < 0.01$ from irinotecan single treatment.

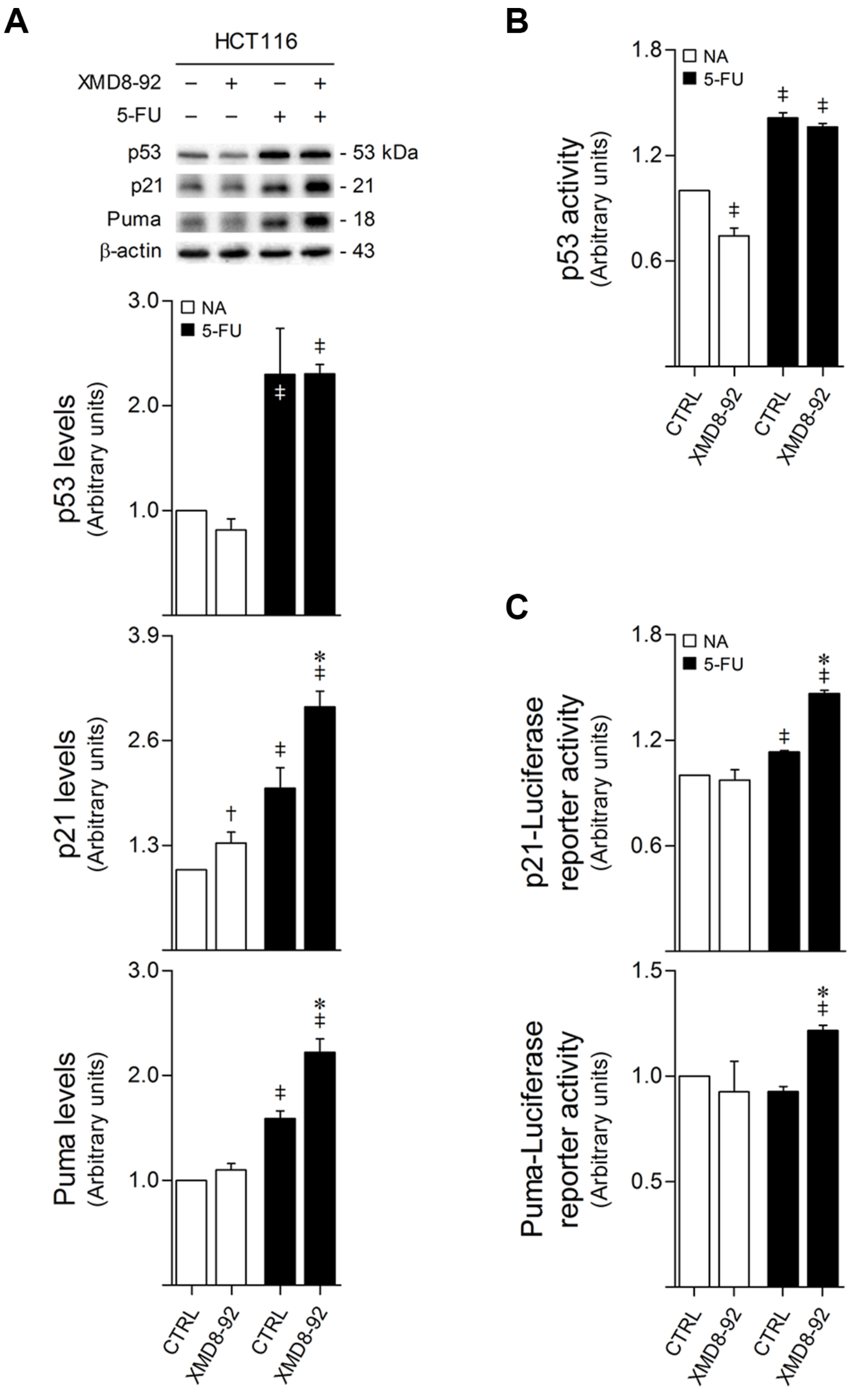

Supplementary Figure S4: ERK5 inhibition with XMD8-92 induces p53 transcriptional activation in HCT116 cells. HCT116 cells were incubated with either 8 μM 5-FU, 4 μM XMD8-92, or both. DMSO was used as vehicle control. (**A**) At 24 h after treatment, cells were harvested for total protein extraction. Protein expression levels were evaluated by western blot. Representative blots are shown. (**B**) The DNA-binding capacity of nuclear p53 was measured 24 h following treatment using the TransAM p53 assay. (**C**) p53-dependent transactivation of *p21/WAF1* (p21-Luc, upper panel) or *PUMA* (PUMA-Luc, lower panel) promoter-reporter constructs was determined at 24 h following treatment. Results are expressed as mean ± SEM fold-change from vehicle control cells of at least 3 independent experiments. *$p < 0.01$ from 5-FU single treatment; †$p < 0.05$ and ‡$p < 0.01$ from vehicle control cells.
